# Differential Effects of Endothelial Cell- as opposed to Neutrophil-Lineage Restricted PD-L1 Gene Expression on Experimental Murine Shock/ Sepsis-Induced Lung Injury

**DOI:** 10.21203/rs.3.rs-7687015/v1

**Published:** 2025-10-07

**Authors:** Elizabeth W. Tindal, Chun-Shiang Chung, Yaping Chen, Runping Zhu, Alfred Ayala

**Affiliations:** Brown University Health-Rhode Island Hospital; Brown University Health-Rhode Island Hospital; Brown University Health-Rhode Island Hospital; Brown University Health-Rhode Island Hospital; Brown University Health-Rhode Island Hospital

**Keywords:** PD-L1, indirect-acute lung injury, endothelial cells, neutrophils, mice

## Abstract

**Introduction:**

Our laboratory and others have shown that Programmed cell death receptor-Ligand 1 (PD-L1), contributes to the development of shock/ sepsis induced morbidity/ mortality, but its role appears to vary across organ/cell type.

**Objective:**

Here we leverage the construction of Cre-lox mouse models to produce mice constitutively lacking either PD-L1 gene expression on endothelial cells (*ec*PD-L1^−/−^) or neutrophils (*pmn*PD-L1^−/−^), respectively, to test the hypothesis that endothelial cell as opposed to neutrophil deficiency PD-L1 differentially contributes to shock/ sepsis induced lung injury/ death.

**Methods:**

Adult male C57BL/6 (WT), *ec*PD-L1^−/−^, *pmn*PD-L1^−/−^ and/or mixed ^flox^-no cre (Control) mice were subjected to either hemorrhagic (Hem) shock followed 24 hrs by cecal ligation & puncture (CLP) (Hem/CLP) or sham Hem and sham CLP (Sham). Survival studies were done. A separate set of animals were taken at 24 hrs post-procedure for peripheral blood, broncho-alveolar lavage fluid (BALF), lung tissues were harvested, processed/ stained for flow cytometry, cytokine/ chemokine/ angiopoietin ELISAs and indices of organ injury assays. A subset of animals was also examined for changes in lung permeability using Evan’s Blue dye exclusion.

**Results:**

14-day mortality in the *ec*PD-L1^−/−^ mice was lower than in the Hem/CLP Control group, while the mortality rate was increased in the *pmn*PD-L1^−/−^ vs. Controls. Lung vascular permeability was also markedly decreased in the *ec*PD-L1^−/−^ Hem/CLP mice but no such decline was seen in the lungs of *pmn*PD-L1^−/−^ mice. While Hem/CLP increased the lung tissue, BALF and blood levels of several cytokine/ chemokine/angiopoietin levels, the concentrations of lung tissue, BALF MCP-1 and blood BUN markedly declined in the *ec*PD-L1^−/−^ vs. Control mice. Alternatively, the lung levels of Angiopoietin-2 and BALF MIP-2 and IL-6 concentrations significantly increased in Hem/CLP *pmn*PD-L1^−/−^ animals.

**Conclusions:**

Taken together, these results support the hypothesis we have previously proffered that expression of PD-L1 on endothelial cells has a morbid impact. However, surprisingly, we have also uncovered a potential immune protective role of PD-L1 expression on neutrophils.

## Introduction

Traumatic injury, of which rapid and excessive blood loss is often a significant component, remains a major concern for healthcare providers worldwide as it is highly associated with clinical morbidities [1; 2; 3; 4]. The timing following injury by which clinicians can rapidly treat and resuscitate these individuals is tightly linked to patient survival. However, while trauma care directed toward rapid patient stabilization and support continues to advance, there remain a variety of comorbidities concurrent with hypovolemic shock that still present significant challenges to successful treatment, despite the best efforts of clinicians [5; 6]. Of these morbidities - the development of sepsis is particularly detrimental to survival. “Sepsis” is now defined as a life-threatening organ dysfunction caused by a dysregulated host response to infection (Sepsis 3) [7]. Those end-organ dysfunctions can consist of the lungs, heart, liver, kidney, gut, brain, etc. Relative to the host response to injury/infection, sepsis is proposed to result from a multi-factorial disharmony of immune cell function, in which circulating immune cell influx into distal organs, contributing directly or indirectly to tissue/organ injury [8; 9]. Most frustrating is that while we continue to optimize the supportive care for these critically ill/injured patients, we have yet to see a novel molecular etiology-based therapy make a sustained impact on overall septic morbidity/mortality [10; 11]. And while one can have debates about trial design [12; 13; 14], pre-clinical study quality [15; 16; 17] and other aspects of clinical application of novel agents [18], the need remains to better understand the mechanistic complexity of shock and/or sepsis in humans/animals if we are to ever translate new knowledge into patient diagnostics and therapies [18; 19].

Looking more closely at lung injury. This can be the result of a ‘primary/direct’ pulmonary process such as pneumonia, to aspiration or some other injury/issue that has its derivation from the lung itself. Or it can result ‘indirectly’ from a distal inflammatory and/or infectious process located elsewhere in the body as is the case in traumatic shock/injury, non-pulmonary sepsis, pancreatitis, etc. The most severe form of either is defined as acute respiratory distress syndrome (ARDS) and presents with hypoxemia and radiological infiltrates/fluid build-up/edema on chest X-ray [20]. Unfortunately, while much has been learned over the years about the pathomechanisms of acute lung injury/ARDS [21], there is still uncertainty as to what drives the development of ‘indirect’ acute lung injury resulting from shock and/or sepsis that has contributed to the inability to develop etiology/pathologically driven therapeutics. There are two proposed theories: 1st that ‘primed’ neutrophils migrate to lung, where they reside until subsequently triggered to release their bactericidal/inflammatory cargos, when triggered by a secondary stimulus, culminating in “by-stander” lung tissue damage [22; 23; 24; 25]. The 2nd proposes that local pulmonary immune cell activation predisposes epithelial and endothelial cell dysfunction which drives ‘indirect’ ARDS [25; 26; 27; 28].

That said, while working for years on defining numerous defects of components of both adaptive and innate immune responsiveness induced by experimental shock and/or sepsis, our laboratory has uncovered novel role(s) for a number of the members of the B7-family of cell-associated co-inhibitory receptors, i.e., Programmed Cell Death Receptor-1 [PD-1], B-/T-Lymphocyte Attenuator [BTLA], recently, V-domain Immunoglobulin Suppressor of T cell Activation [VISTA, a.k.a., B7-H5, PD-1H] and their respective cell surface ligands; popularly referred to as checkpoint proteins, that appear to contribute to aspects of organ and/or lung injury [29]. Classically the function of PD-1 and PD-L1 have been defined based on their function/interactions with T cells during the process of antigen presentation and subsequent T cell activation. In this respect, for full activation of the T cell to occur upon exposure to foreign pathogenic and/or tumor antigen via presentation by the major histocompatibility antigen to the T Cell Receptor (signal 1), the T cell requires a 2nd signal, a sort of stop or go signal in the form of these immune checkpoint proteins that serve to co-stimulate or co-inhibit its activation. Exploring prior research from our lab, we have reported that in model of traumatic shock and subsequent septic challenge, as produced using experimental hemorrhage and cecal ligation and puncture (CLP) that global PD-L1 knockout mice significantly improved survival when compared to their wildtype counterparts [30]. Further, these mice did not develop acute lung injury as evidenced by their protein levels in bronchoalveolar lavage fluids. Also, at the whole lung tissue level knockout mice maintained, as evidence by western blot analysis, their endothelial integrity in the face of trauma and sepsis through stable expression of VE cadherin as well as an increase in zona occludens expression. And while there was decreased neutrophil chemotaxis to the lungs there was no decline in associate MPO activity detected. Locally we saw a reduction in lung tissue cytokine levels including TNF-α, IL-6 and MCP-1. Tying this all together, we speculated that PD-L1 expression on endothelial cells might be having a negative impact with increased permeability, a reduction in growth factors and loss of junctional integrity. Systemically, increased PD-L1 expression appears to be tied to an increased organ damage, cytokine release and death in response to the combined insults of shock & sepsis. However, while leaning towards a pathological role for PD-L1 induced expression by endothelial cells in the morbidity/ mortality that has been previously reported [31], the approaches in these studies cannot rule out a role for PD-L1 expression on leukocytes like neutrophils.

To get at the question of how does expression of PD-L1 on endothelial cells as opposed vs. leukocytes, specifically neutrophils, affect the on-set of shock/septic lung iALI/iARDS and/or overall morbidity/mortality, we chose to build a endothelial cell lineage as opposed to a neutrophil cell lineage specific mice that are constitutively deficient for the PD-L1 gene as opposed the global PD-L1 gene knockout animals to test the hypothesis that cell-lineage selective loss of PD-L1 gene expression will differentially contribute to shock/septic iARDS and/or overall morbidity/ mortality.

## Material and Methods

### 2.1 Mice

Male C57BL/6 mice were purchased from The Jackson Laboratory (Bar Harbor, ME). Animals obtained from our outside vendor were acclimated no less than 7 days, and often longer [maximum ~5 weeks], prior to utilizing these animals in the studies described here. During this period, they were housed in the Rhode Island Hospital (RIH) rodent facility (12-hours: 12-hours light/dark cycle, 23–25°C, 30–70% humidity) where they received standard care and diet (standard rodent chow)/water *ad libitum*. All protocols were carried out in the morning (8–11AM) and were performed in accordance with the National Institutes of Health guidelines and as approved by the Animal Use Committee of Rhode Island Hospital (AWC# 5054–21 & 5028–24). PD-L1[CD274/B7-H1]^loxP^ mouse (PD-L1^fl/fl^) was derived from cryopreserved embryo at the Taconic Knockout Repository (mouse TF0103 [MGI:1926446]) at the Taconic Biosciences Inc. (Germantown, NY), which had LoxP sites inserted by targeted mutation at chromosome 19.28620055–28640695 gene NM_021893. The VE-Cadherin-Cre (Strain #:006137; RRID:IMSR_JAX:006137) and the S100a8-Cre (Strain #:021614; RRID:IMSR_JAX:021614) breeder mouse strains were also obtained from The Jackson Laboratory.

### 2.2 Endothelial cell or neutrophil restricted constitutive PD-L1 gene deficient mice

Initial genotyping strategy and PCR results for F0 and F1 generations are described in Supplemental Figure 1 and 2, for the establishment of the PD-L1^flox/flox^ VE-Cadherin-Cre mice (*ec*PD-L1^−/−^) and the PD-L1^flox/flox^ S100a8-Cre mice (*pmn*PD-L1^−/−^), respectively, as well as for delineating/establishing the PD-L1^flox/flox^ homozygous breeder mice from PD-L1^WT/flox^ or PD-L1^WT/WT^ animals (Controls). Routine genotyping of the PD-L1 gene expression, with [^WT^ or ^WT/flox^]/ without the LoxP insertion [^flox/flox^]; the VECadherin-Cre transgene; and S100a8-Cre transgene mice were performed on tail biopsy samples collected after weaning [32]. Tail samples were processed for PCR (primers described below) and treated with custom 25nmole DNA oligos from Integrated DNA Technologies (Coralville, IA). Following PCR amplification, samples were run on a SDS page gel and imaged for gene deletion analysis and validation. Male mice with appropriate base pair deletion were used for downstream studies. All mice were housed, bred, and maintained at the Rhode Island Hospital Central Research Facilities.

Complementary (c)DNA was synthesized via reverse transcription of RNA, and primer pairs (Integrated DNA Technologies) for the PD-L1 gene were: sense, 5’-GAA GCT TTG CCT AAA GCA GG-3’ and antisense, 5’-GTC TGG AAA GAG CAG ACG AG-3’as described/designed by Taconic Biosciences Inc.; for VE-Cadherin-Cre transgene: sense, 5′-GTG AAA CAG CAT TGC TGT CAC TT-3′ and antisense, 5′-GCG GTC TGG CAG TAA AAA CTA TC-3′; and for S100a8-Cre transgene: sense, 5′-GTG AAA CAG CAT TGC TGT CAC TT-3′ and antisense, 5′-GCG GTC TGG CAG TAA AAA CTA TC-3′ both as described/designed by The Jackson Laboratory.

Flow cytometric analysis of single cell lung tissue specimens or the analysis peripheral blood leukocytes isolated form either ‘genotyped’ *ec*PD-L1^−/−^, *pmn*PD-L1^−/−^ or Control animals was also done to document the PD-L1 endothelial cell restricted phenotypic deficiency or the PD-L1 neutrophil restricted phenotypic deficiency of either the *ec*PD-L1^−/−^ or *pmn*PD-L1^−/−^ animals, respectively, as compared to ‘Control’ mice (Supplemental Figure 3A-D). Details of the actual staining protocol for flow cytometric analysis are described below.

### 2.3 Hemorrhage/CLP model [33]

A model of hypovolemic shock (Hem) was implemented, whereby a fixed-pressure hemorrhage was utilized to achieve a sustained reduced mean arterial blood pressure in C57BL/6, various incomplete floxed-Cre background control (control) or *ec*PD-L1^−/−^ or *pmn*PD-L1^−/−^ male mouse hosts [33]. This choice was made so as to maximize our ability to initially see an experimental difference in the ALI/ARDS response based on previous reports that male mice did poorer in response to these experimental stressors of shock (hemorrhage) and/or septic challenge than pro-estrus stratified females [34; 35]. In brief, an isoflurane/oxygen gas mixture was used as anesthesia under the Rhode Island Hospital IACUC approved protocol for animal safety (AWC# 5054–21 & 5028–24). Bi-lateral arteriotomies were catheterized and used to monitor blood pressure and draw blood throughout the procedure. Mice were kept in this state for a 90-minute duration, during which additional blood was drawn to maintain reduced blood pressure ~40mmHg (+/− 5mmHg). This provides a standardized and effective mimic of severely injured hypovolemic patients. Immediately following this experimental insult, mice were administered a crystalloid solution of lactated ringer’s equivalent to 4X the volume of blood hemorrhaged. Sham surgeries were performed under anesthesia by which both femoral arteries were ligated. However, blood was not drawn, and these serve as negative controls in this analysis.

Subsequently, cecal ligation and puncture (CLP) as described previously [33] was performed at 24 hours post-Hem/Post-Sham Hem (Sham) on mice. In brief, following midline laparotomy, the cecum was ligated ~1 cm above the cecal tip and punctured twice with a 22G needle. Cecal contents were extruded into the intraperitoneal cavity. The abdomen was closed using a sterile PDO suture. Mice were treated with lidocaine on the muscle layer and a subcutaneous injection of 1mL Lactated Ringer’s solution. Mice were euthanized 24-hours post procedure to isolate various tissues for downstream studies or the animal’s survival was followed for 14-days (Supplemental Figure 4).

### 2.4 Sample Acquisition

Blood/plasma, bronchoalveolar lavage fluids (BALF) and lung tissues were collected at 24 h post-CLP when sequential Hem/CLP model was performed to assess Tie 2, Ang-1/Ang-2, cytokine/chemokine, Evans blue dye content (as a measurement for pulmonary vascular leak), and AST/ALT as an index of liver injury and BUN as an index of kidney injury [33; 36; 37].

### 2.5 Flow cytometry

#### 2.5.1 Mouse cell phenotyping

The lungs were harvested from mice 24-hours following sham or Hem/CLP procedure. The lungs were homogenized using frosted slides and red blood cells were lysed using a Na^+^Cl^−^ gradient. Briefly, single cell suspensions of lung tissue were prepared using a mouse lung dissociation kit (Miltenyi biotec.; Cat#: 130–095-927)and gentleMACS^™^ dissociator as per manufacturer’s protocol (Miltenyi biotec, Auburn, CA). Neutrophil and endothelial cell (EC) expression of PD-L1 was analyzed for Sham Controls or *ec*PD-L1^−/−^ or *pmn*PD-L1^−/−^ mouse single-cell suspensions of saline-perfused lung tissue as previously described [30]. In brief, based on forward/side scatter results for lung ECs, the granulocyte population was gated out. Neutrophils were characterized as Ly6G^+^ while endothelial cells were characterized by CD31^+^. Expression of PD-L1 was calculated as %PD-L1^+^Ly6G^+^ or %PD-L1^+^CD31^+^ in lung tissue and were determined using a Miltenyi-MACSQuant Analyzer 10 (Miltenyi biotec, Auburn, CA). To compensate for spectral overlap, UltraComp eBeads Plus Compensation Beads (ThermoFisher Scientific: cat# 01–3333-41) were used according to manufacturer’s protocol. Fluorescence minus one (FMO) controls were used to determine positive expression gates during analysis using FlowJo software (FlowJo LLC, Ashland, OR). Fluorochrome conjugated antibodies used: anti-Ly6G (clone 1A8), anti-B7-H1/PD-L1 (clone MIH5) (R&D Systems, Minneapolis, MN) and anti-CD31 (clone 390) (BD Bioscience, San Diego, CA).

### 2.6 Assessment of lung capillary leakage by extravasation of Evans blue (EB) dye method.

Evidence of change in vascular integrity of the lungs was sought by assessing the extent of EB dye extravasation [38] in a separate set of animals. To assess this, mice were administered 20mg EB/kg body weight via their tail vein. 30 minutes later, the mice were anesthetized with isoflurane, a 0.6 ml blood plasma sample was taken, the mice were exsanguinated, and the lungs were instilled with 2.0 ml volume of the PBS. Following overnight extraction, the tissue was centrifuged at 3,000X g. EB concentration in the tissue supernatant was determined spectrophotometrically at 620 nm prepared in formamide. Lavage content is expressed of the plasma concentration, and tissue content of EB is expressed as micrograms/mg dried lung tissue.

### 2.7 Cytokine, Chemokine & Angiopoietin related protein analysis

Plasma, BALF and lung tissue lysates samples were collected and stored as described in previous section [33; 36; 37]. To assess cytokine concentration in these samples the following ELISA kits were used according to manufacturer’s instruction as we have previously reported [39; 40; 41]: ELISA MAX Standard Sets for mouse IL-6 (BioLegend, cat# 431301), IL-10 (BioLegend, cat# 431411), TNF-α (BioLegend, cat# 430901) and MCP-1 (BioLegend, cat# 432701); R & D Systems Duo Set Mouse ELISA kits for CXCL2/MIP-2 (R & D Systems, cat# DY452), CXCL1/KC (R & D Systems, cat# DY453) and Tie −2 (R & D Systems, cat# MTE200); MyBioSource ELISA kit for Mouse Angiopoietin 1 (MyBioSource, cat# MBS727480) as well as Abcam Mouse Angiopoietin 2 ELISA (Abcam, cat# ab171335).

### 2.8 Colorimetric assays for organ morbidity

To assess indices of tissue injury, blood was collected from mice 24-hours following sham-Hem/CLP or Hem/CLP procedure via cardiac puncture using a heparin coated syringe. Blood sample was centrifuged at 10,000rpm and supernatant (plasma) was collected and stored at −80°C. For tissue injury assays, plasma was analyzed using the following kits according to manufacturer’s protocol: Urea Nitrogen (BUN) Colorimetric Detection Kit (Invitrogen cat# EIABUN), Alanine Aminotransferase (ALT) Activity Assay Kit (Sigma Aldrich: cat# MAK052), and Aspartate Aminotransferase (AST) Activity Assay Kit (Sigma Aldrich: cat# MAK055).

### 2.9 Statistical analysis

Statistically significant differences between multiple groups were determined using a Kruskal Wallis test or by a Mann-Whittney-U test for 2 groups only for nonparametric data. Survival data was presented as a Kaplan-Meier curve and the presence of statistically significant difference between groups was established with a Log-rank survival test. Alpha was set to 0.05 as the cut off for statistical significance using Prism 9.3.0 (GraphPad Software) statistical software. Summary data is provided as data dot-plot overlaying histograms showing the group mean and standard deviation.

## Results

### 3.1 Selective vascular endothelial PD-L1 gene deficiency is associated with improved survival following Hem/CLP, but not selective neutrophil PD-L1 gene loss (these animals exhibited poorer overall survival).

**Figure 1.**
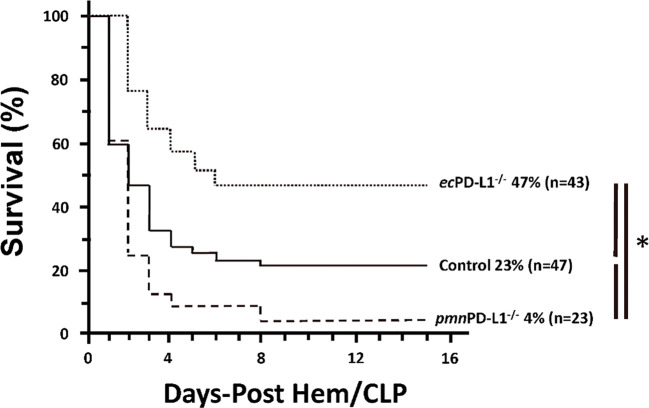
Selective vascular endothelial PD-L1 gene deficiency is associated with improved survival following Hem/CLP, but not selective neutrophil PD-L1 gene loss (these animals exhibited poor overall survival vs. Controls). Control, *ec*PD-L1^−/−^ or *pmn*PD-L1^−/−^ mouse 14-day overall survival after subjecting them to the sequential insults of Hem/CLP as depicted by Kaplan-Meier curves. The n/treatment group is listed in parentheses; the presence of a significance difference between groups was established at * p<0.05 by a Log-Rank test.

With respect to our initial question of whether endothelial cell restricted gene deficiency PD-L1 would have protective effect on overall mortality the sequential exposure to hemorrhagic shock followed experimental septic challenge/ CLP, we observed the overall survival of *ec*PD-L1^−/−^ mice was markedly increased over Control animals (see Figure 1). Alternatively, we actually detected an overall decline in survival of *pmn*PD-L1^−/−^ mice when compared again to the Control animals. Of note we also look at the septic mortality of our Control animals as opposed to C57BL/6 mice obtained from Jackson Laboratories and could see no significant difference in the survival of the animals over 14-days of study (Supplemental Figure 5). Inasmuch, we have not included C57BL/6 mice and only utilized our in-house bred Control and for all the residual studies described here relative to the *ec*PD-L1^−/−^ or *pmn*PD-L1^−/−^ animals.

### 3.2 The lung vascular permeability is markedly decreased in the vascular endothelial PD-L1 gene deficient Hem/CLP mice, however, no such decline in permeability was seen in the lungs of mice lacking PD-L1 gene expression restricted to neutrophils.

**Figure 2.**
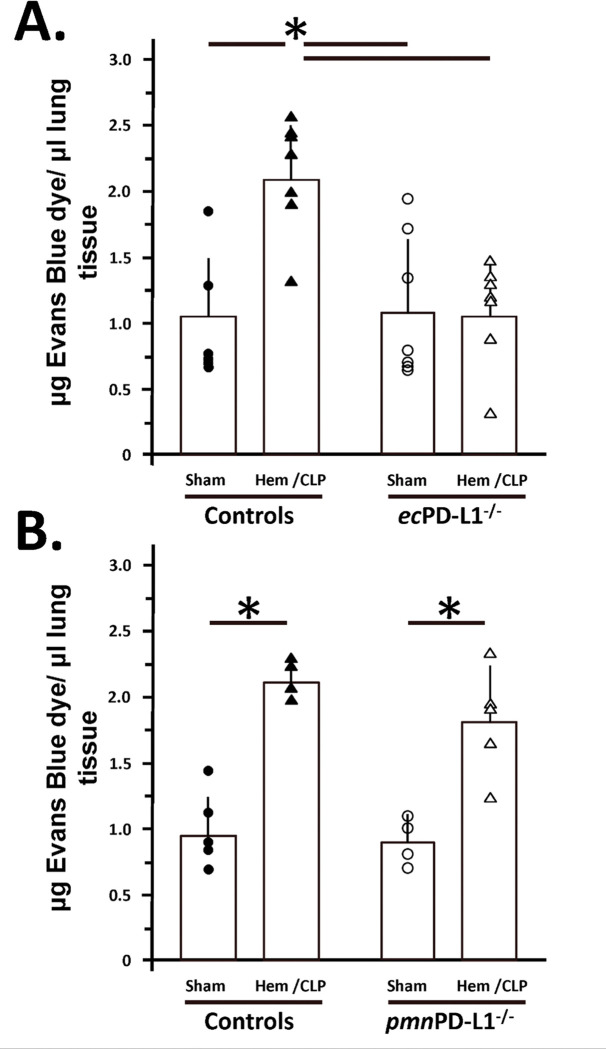
Endothelial cell as opposed to neutrophil restricted PD-L1 gene deficiency reduced evidence of pulmonary vascular leak seen 24 hours after exposure to the sequential insults of experimental shock/ sepsis when compared to their gene replete equivalent Controls. The assessment of lung capillary leakage was done by extravasation of Evans blue (EB) dye method. The n/treatment group are shown as symbols super-imposed on histogram depicting the group mean ± the standard deviation; the presence of a significance difference between groups was established at * p<0.05 by a Kruskal-Wallis multiple comparisons test.

As an index of pulmonary dysfunction as well as an index of vascular endothelial barrier permeability we chose to look at the ability to exclude intravenously delivered Evan’s Blue dye from tissue. In Control animals (Figure 2A & B) subjected to Hem/CLP, as well as C57BL/6 background mice (see Supplemental Figure 5A), there was significantly and consistent increase in lung permeability. Importantly, we found that the rise lung vascular permeability was also markedly attenuated in the *ec*PD-L1^−/−^ Hem/CLP mice when compared to Control Hem/CLP animals (see Figure 2A), while no such decline in permeability was seen in the lungs of *pmn*PD-L1^−/−^ mice (see Figure 2B).

### 1.1 Hem/CLP selectively, attenuated BALF and lung tissue, cytokine/chemokine levels (specifically, MCP-1 and to a lesser extent MIP-1) in the vascular endothelial cell PD-L1 gene deficient mice as opposed to mice lacking PD-L1 gene expression selectively on their neutrophils (which tended to potentiate and/or not alter the elevated MCP-1, MIP-2 mediator levels).

**Figure 3.**
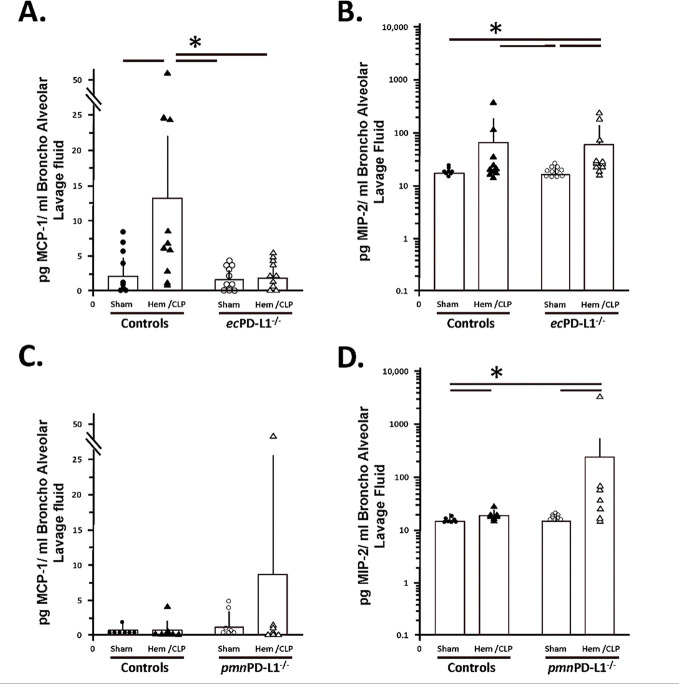
Hem/CLP selectively, attenuated BALF chemokine levels, e.g., MCP-1, in the vascular endothelial cell PD-L1 gene deficient mice as opposed to mice lacking PD-L1 gene expression selectively on their neutrophils (which tended to potentiate, the Hem/CLP elevated MCP-1 and MIP-2 levels). BALF chemokine levels were established by ELISA. The n/treatment group are shown as symbols super-imposed on histogram depicting the group mean ± the standard deviation; the presence of a significance difference between groups was established at * p<0.05 by a Kruskal-Wallis multiple comparisons test.

**Figure 4.**
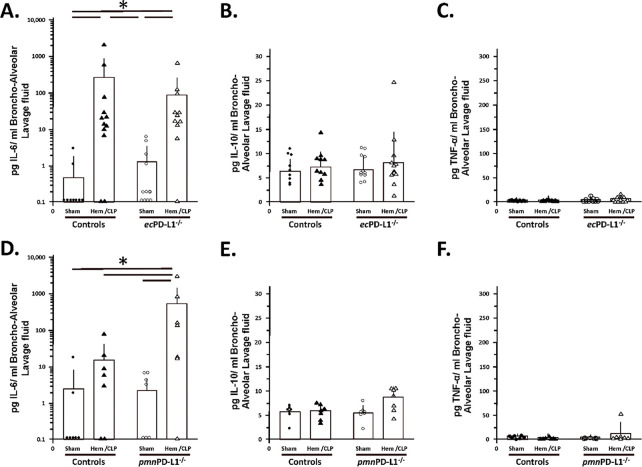
Hem/CLP selectively altered BALF cytokine levels in the vascular endothelial PD-L1 gene deficient mice as opposed to mice lacking PD-L1 gene expression selectively on their neutrophils, e.g., potentiated IL-6 levels above Hem.CLP Controls. BALF cytokine levels were established by ELISA. The n/treatment group are shown as symbols super-imposed on histogram depicting the group mean ± the standard deviation; the presence of a significance difference between groups was established at * p<0.05 by a Kruskal-Wallis multiple comparisons test.

**Figure 5.**
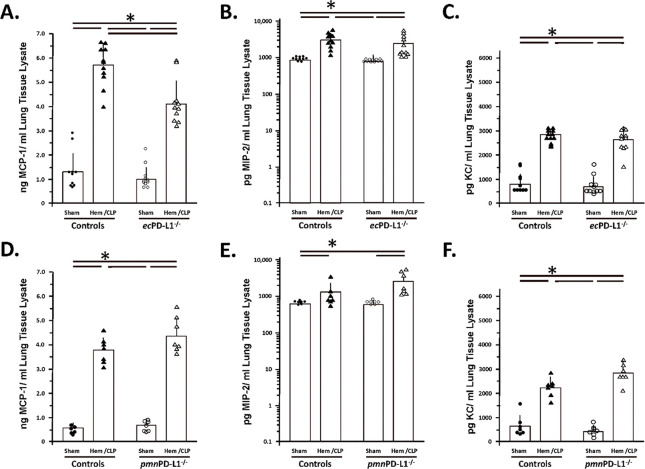
Hem/CLP selectively altered lung tissue chemokine levels in the vascular endothelial PD-L1 gene deficient mice, e.g., attenuated MCP-1 levels, as opposed to mice lacking PD-L1 gene expression selectively on their neutrophils. Lung tissue lysate chemokine levels were established by ELISA. The n/treatment group are shown as symbols super-imposed on histogram depicting the group mean ± the standard deviation; the presence of a significance difference between groups was established at * p<0.05 by a Kruskal-Wallis multiple comparisons test.

**Figure 6.**
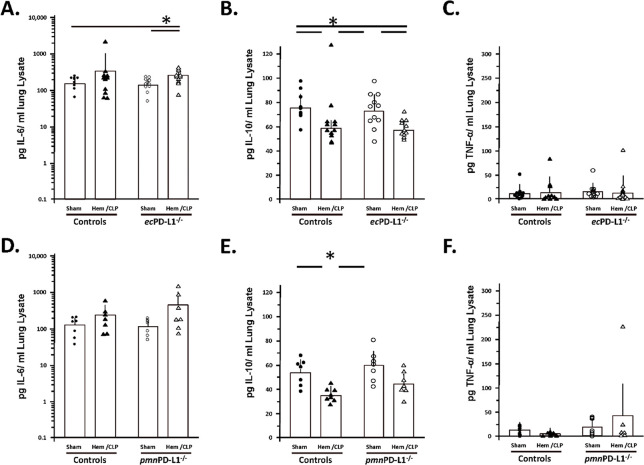
Hem/CLP altered lung tissue cytokine levels, i.e., IL-6, IL-10 and TNF-α, but this was not markedly affected by either vascular endothelial or neutrophil-restricted loss of PD-L1 gene expression. Lung tissue lysate cytokine levels were established by ELISA. The n/treatment group are shown as symbols super-imposed on histogram depicting the group mean ± the standard deviation; the presence of a significance difference between groups was established at * p<0.05 by a Kruskal-Wallis multiple comparisons test.

While subjecting Control group mice to Hem/CLP or in most cases the C57BL/6 background mice (see Supplemental Figure 6B-E), typically significantly increased their level of MCP-1, MIP-2 and IL-6 detected in BALF of the *pmn*PD-L1^−/−^ mice, no change was observed in BALF TNF-α or IL-10 (see Figures 3 & 4). Of those changes induced by Hem/CLP, the rise in MCP-1 was only attenuated in *ec*PD-L1^−/−^ mice (see. Figure 4A). Relative to these cytokine/chemokine levels in the lung tissue itself, 24 hours following Hem/CLP Control mouse MCP-1, MIP-2 and KC were seen consistently to rise (see Figure 5A-F) which was comparable to the C57BL/6 background mice (see Supplemental Figure 5F-H). Alternatively, unlike the C57BL/6 mice were Hem/CLP induced a marked rise in both lung tissue lysate IL-6 and TNF-α levels (see Supplemental Figures 5I, K), while trending higher, no statistically significant changes were observed in IL-6 as well as TNF-α in Hem/CLP *ec*PD-L1^−/−^ or *pmn*PD-L1^−/−^ as opposed to their respective Control animals (Figure 6A, C, D & F). However, there was a consistent marked decline in the Control mouse IL-10 levels following Hem/CLP of either *ec*PD-L1^−/−^ or *pmn*PD-L1^−/−^ (see Figure 6B & E), which in one the few discordances were unchanged in the C57BL/6 background mice (see Supplemental Figure 6J). Also, of those cytokines/ chemokines increased in the Hem/CLP Control mouse lungs only the levels of IL-6 in the Hem/CLP *ec*PD-L1^−/−^ mice lungs were suppressed (see Figure 6A).

### 1.2 Hem/CLP induced-changes in lung tissue Angiopoietin 1, 2 and Tie-2 levels were attenuated in the neutrophil-restricted PD-L1 gene deficient mice as opposed to mice lacking PD-L1 gene expression on their vascular endothelial cell.

**Figure 7.**
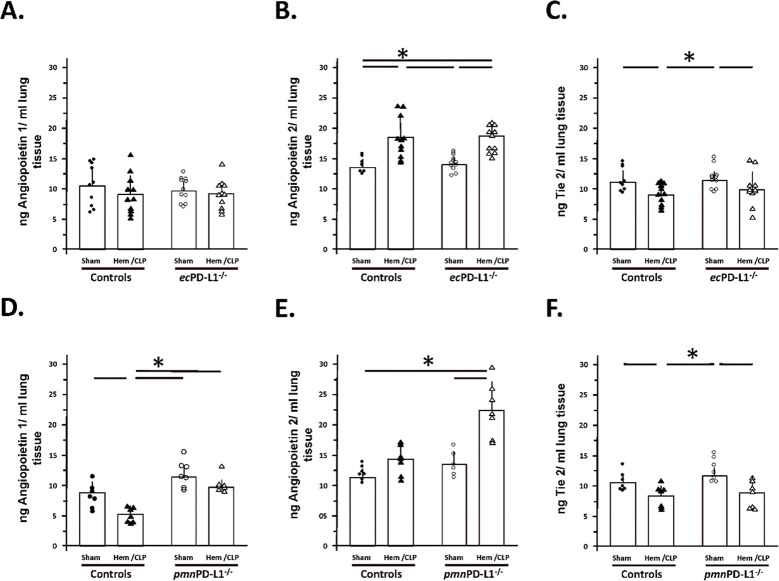
Hem/CLP induced-changes in lung tissue Angiopoietin 1, 2 and Tie-2 levels were attenuated in the neutrophil-restricted PD-L1 gene deficient mice as opposed to mice lacking PD-L1 gene expression on their vascular endothelial cell. Lung tissue lysate growth factor levels were established by ELISA. The n/treatment group are shown as symbols super-imposed on histogram depicting the group mean ± the standard deviation; the presence of a significance difference between groups was established at * p<0.05 by a Kruskal-Wallis multiple comparisons test.

Relative to the lung tissue levels of Angiopoietin 1 these were unchanged in Sham vs. Hem/CLP groups in both the Control or *ec*PD-L1^−/−^ mice (see Figure 7A) (This was also the case for the C57BL/6 background animals; see Supplemental Figure 6L), however, a marked decline was noted in Angiopoietin 1 concentration in the Control Hem/CLP animals that was not evident in the Hem/CLP the *pmn*PD-L1^−/−^ mice when compared to their respective Sham groups (see Figure 7D). Here again, in Control group mice subjected to Hem/CLP 24 hours earlier there was a marked rise in lung tissue Angiopoietin 2 concentration, which was not changed in *ec*PD-L1^−/−^ mice (Figure 7B) (This was also the case for the C57BL/6 background animals; see Supplemental Figure 5M). Alternatively, when *pmn*PD-L1^−/−^ mice were subjected to Hem/CLP (see Figure 7E) the levels of Angiopoietin 2 were markedly elevated above both the Sham or Hem/CLP Control mouse groups as well as the equivalent Sham *pmn*PD-L1^−/−^ group. With respect to Tie-2 levels, these were significantly lower in the animals subjected to Hem/CLP (The same change was also seen for the C57BL/6 background animals; see Supplemental Figure 6N), however, this did not change irrespective if they were from the Control, *ec*PD-L1^−/−^ or the *pmn*PD-L1^−/−^ mouse background (see figure 7C & F).

### 1.3 Systemic blood cytokine/chemokine levels while typically elevated by Hem/CLP were not markedly altered by selective endothelial cell or neutrophil loss of PD-L1 gene expression.

**Figure 8.**
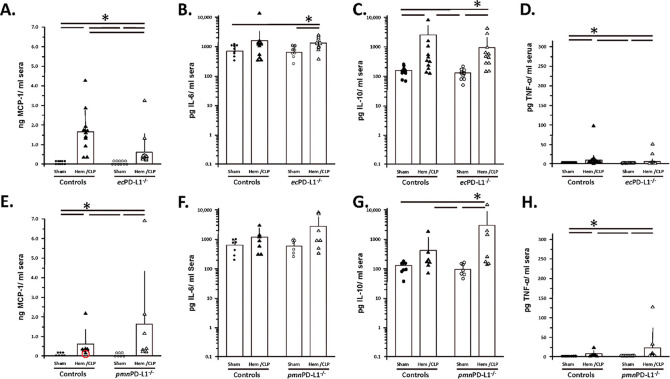
Hem/CLP selectively, attenuated MCP-1 levels in the vascular endothelial cell PD-L1 gene deficient mice as opposed to mice lacking PD-L1 gene expression, which tended toward elevated MCP-1 and IL-10 levels, when compared with Controls systemic chemokine/cytokine concentrations. Systemic blood plasma chemokine/cytokine levels were established by ELISA. The n/treatment group are shown as symbols super-imposed on histogram depicting the group mean ± the standard deviation; the presence of a significance difference between groups was established at * p<0.05 by a Kruskal-Wallis multiple comparisons test.

An examination of the systemic blood/plasma cytokine levels evident at 24 hours post-surgical protocol documented, as previously reported by our laboratory [30; 31] and as documented in the C57BL/6 background animals (see Supplemental Figure 7A-D), that Hem/CLP induces a marked rise in cytokines/chemokines like MCP-1, IL-10 and TNF (see Figure 8A, C, D, E, G & H). Interestingly, there was a statistically significant downward and a non-statistically upward trend in MCP-1 levels exhibited by Hem/CLP *ec*PD-L1^−/−^ or the *pmn*PD-L1^−/−^ mice, respectively, when compared to either Sham or Hem/CLP Control group animals (see Figure 8B & F). Somewhat, surprisingly, while again an upward trend in plasma IL-6 levels in the various Hem/CLP mouse groups was seen when compared to their respective Sham group this did not reach statistical significance, with the exception Hem/CLP *ec*PD-L1^−/−^ vs it’s respective Sham group (see Figures 8B & F).

### 1.4 Blood plasma indices of kidney and liver injury/dysfunction were consistently elevated Hem/CLP mice irrespective of whether they were derived from endothelial or neutrophil PD-L1 gene deficient animals.

**Figure 9.**
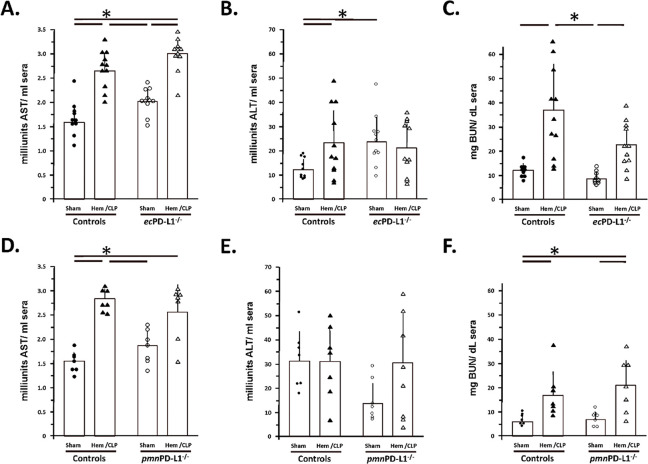
Blood plasma indices of kidney and liver injury/dysfunction were consistently elevated Hem/CLP mice irrespective of whether they were derived from endothelial or neutrophil PD-L1 gene deficient animals. AST, ALT and BUN levels were established by commercial assay. The n/treatment group are shown as symbols super-imposed on histogram depicting the group mean ± the standard deviation; the presence of a significance difference between groups was established at * p<0.05 by a Kruskal-Wallis multiple comparisons test.

Finally, while we detected presence of elevated levels of AST and increased BUN in all the Hem/CLP mice sera when compared to their respective Sham groups, we did not see a change in the plasma ALT levels in these same animals (Figure 9A-F; Supplemental Figure 7E-G). This also did not appear to be affected by whether the animals were *ec*PD-L1^−/−^ or *pmn*PD-L1^−/−^ or their respective Controls.

## Discussion

### 2.1 The results of subjecting our *ec*PD-L1^−/−^ mouse model to the sequential insults of Hem/CLP support the hypothesis we have previously proffered that increased expression of PD-L1 on endothelial cells has a morbid impact in the lungs and overall survival.

As already alluded to earlier, our laboratory and others have shown that PD-1 and its best-known ligand, PD-L1, contribute to the development of shock/ sepsis induced morbidity/ mortality due in part to pathological interactions between myeloid cells, e.g., neutrophils, macrophages, dendritic cells, etc., or non-immune cells, such as vascular endothelial cells [30; 31; 39; 40; 42; 43; 44]. However, studies utilizing global gene deficient animal modeling do not readily permit delineation of the contribution(s) of cell-specific gene expression to the development of shock/ septic organ injury [30; 31]. Here we leveraged the construction of Cre-lox mouse models by crossing an adult PD-L1^flox/flox^ mouse with either VE-Cadherin Cre or S100A8-Cre breeder to produce mice constitutively lacking either PD-L1 gene expression on endothelial cells (*ec*PD-L1^−/−^) or neutrophils (*pmn*PD-L1^−/−^), respectively. We then utilized these animals to test the hypothesis that endothelial cell as opposed to neutrophil deficiency PD-L1 gene expression contributes to shock/ sepsis induced lung injury/ death. The studies with *ec*PD-L1^−/−^ mice indicated that selective loss of PD-L1 expression was associated with a marked reduction in lung vascular permeability and produced an improvement in overall survival over 14-days post-insult. This is also in keeping with the assertion we had made in a prior study by Xu et al [31] that had compared the somewhat selective differences in the cell populations that are affected by distinct route of anti-PD-L1 siRNA delivery and/or the nature of the vehicle delivery system. In that experiment while we were able to largely rule out pulmonary epithelial cells targeted sites to account for anti-PD-L1 siRNA shock/septic mouse lung protection, we could not cleanly say that PD-L1 gene expression induced by Hem/CLP was just due to selective targeting of vascular endothelial cells (when the anti-PD-L1 siRNA is liposomally encapsulated and given intravenously) as opposed circulating myelocytes/leukocytes, which would also be impacted by the mode of siRNA administration. Here, the lack of such a protective effect on lung morbidity/ overall mortality in the *pmn*PD-L1^−/−^ as opposed to the protection seen in the *ec*PD-L1^−/−^ animals supports the assertion that this is an endothelial cell mediated effect.

Relative to what might have accounted for this lung protective activity in the *ec*PD-L1^−/−^ mice, we also examined these animals for changes in local, BALF & lung tissue, lysate levels of pro-/anti-inflammatory chemokines/cytokines, angiogenic factors; systemic pro-/anti-inflammatory chemokines/cytokines as well as a few select distal systemic blood indices of liver and/or kidney injury. Surprisingly, the most consistent Hem/CLP *ec*PD-L1^−/−^ mouse lung tissue/BALF/blood induced change was the suppression/attenuation of the rise in MCP-1 levels. This is interesting as it coincides with an observation we made with the global PD-L1^−/−^ mice, which exhibit a marked attenuation of Hem/CLP-induced MCP-1 blood and lung tissue levels [30; 31]. In this regard, MCP-1, a.k.a., CCL2, is C-C motif chemokine family member that plays a central role in controlling macrophage, dendritic cell (but not neutrophils or eosinophils) and memory T-cell recruitment at sites of inflammation/infection [45; 46; 47]. Also, while MCP-1 is produced by myeloid cells of the macrophage/monocyte lineage [48; 49] it can also be derived from vascular endothelial sources [50; 51]. Thus, this makes MCP-1 an interesting potential regulatory chemokine relative to the development of EC-mediated lung injury. That said, further experiments using either a MCP-1 neutralizing antibody or multi-gene EC-restricted knock-out of PD-L1 and MCP-1 would be needed to more clearly demonstrate the significance of MCP-1 expression here.

How the loss of PD-L1 gene expression on the endothelial cells directly/indirectly acts/signals to induce changes in either endothelial cell monolayer permeability and/or mediator/chemokine release, i.e., MCP-1, is also unknown. Studies from our own lab by Lomas-Neira et al[30], Fallon et al [52] and Wu et al [53] applying the global PD-L1 or PD-1 knockout mice suggested the PD-L1−/− mice exhibit increased junctional protein expression, e.g., zona occludins, VE-cadherin, in the face of Hem/CLP and/or experimental septic challenge. While, alternatively, Tao et al [54] demonstrated that over-expression of PD-L1 in human lung microvascular endothelial cell cultures led not only to less junctional protein expression but increased expression of NLRP3, cleaved-caspase-1, apoptosis-associated speck-like protein containing a CARD (a.k.a.: ASC) and gasdermin D as well as potentiated mitochondrial stress. Together, this points at PD-L1-induced inflammatory pathway activation driving aspects of mitochondrial dysfunction and/or cell death.

### 2.2 Alternatively, the results of subjecting our *pmn*PD-L1^−/−^ mouse model to the sequential insults of Hem/CLP did not support the hypothesis that expression of PD-L1 on neutrophils has a morbid impact in the lungs and overall survival.

Interestingly, a number of studies looking at the change in expression blood leukocytes derived from patients or experimental animals, have indicated that in presence of sepsis clinically and/or in response to experimental septic challenge there is a clear and overt rise in the expression of PD-L1 on neutrophils [55; 56]. Huang et al indicated in the setting of cecal ligation and puncture-induced polymicrobial septic challenge that this elevated expression of PD-L1 by Gr-1^+^ blood leukocytes was associated with higher mortality [40]. Wang et al and Petra et al both documented that increased expression of PD-L1 on circulating peripheral blood from critically septic patients correlated/ associated with increased morbidity/mortality [55; 56]). These groups both further documented that in the *ex vivo* setting PD-L1 neutrophils derived from septic patients could markedly suppress pro-inflammatory cytokine expression/secretion and exhibit restricted migratory responsiveness among other actions. This clearly implies such cells have a potential anti-inflammatory role, which could also be seen as immune suppressive depending on the pathological setting in which it is considered. In this study, we surprisingly found that neutrophil cell lineage selective loss of PD-L1 gene expression not only was not protective against the morbidity/ mortality produced in response to the sequential insults of Hem/CLP (as we saw with the *ec*PD-L1^−/−^ mouse) but that it actually increased overall mortality. Considering, the *ex vivo* observations of enhanced PD-L1^+^ patient neutrophil exhibiting potentiated anti-inflammatory functions, we speculate that here in the lineage selective loss of PD-L1 gene expression in our model has removed an important endogenous break on the inflammatory response to this insult. In this respect it’s worth noting that the sequential insult of Hem/CLP markedly potentiated the *pmn*PD-L1^−/−^ mouse BALF MIP-2 and IL-6 levels over the Control Hem/CLP animals. And while there were trends toward increases in the *pmn*PD-L1^−/−^ Hem/CLP mouse BALF: MCP-1 & IL-10; lung tissue: MCP-1, MIP-2, KC, IL-6 & TNF-α; as well as plasma: MCP-1, IL-6, IL-10 & TNF-α, when comparing it to *ec*PD-L1^−/−^ Hem/CLP mouse levels these were not statistically significant. However, together they seem to speak to the loss of a potential anti-inflammatory function of the *pmn*PD-L1^−/−^ mouse under these conditions. Also, as this is a ‘pre-dispositional’/ ‘constitutive’ neutrophil lineage selective loss of PD-L1 this would most likely have had a temporal impact on the early/acute pro-inflammatory response to these sequential insults of shock/infection.

### 2.3 Selective PD-L1 gene deficiency in *pmn*PD-L1^−/−^ lead to a potentiation of the vascular growth factor Angiopoietin 2 in response to the sequential insult of shock/sepsis.

Another interesting observation is that loss of PD-L1 gene expression on the neutrophil seemed to also markedly increase lung tissue Angiopoietin-2 levels. As neutrophil interaction with the endothelial cell surface has been reported to have a role in inducing the release of pre-stored mediators, of which Angiopoietin-2 is one, from the endothelial cell’s Weibel Palade bodies [36; 57; 58] it’s tempting to speculate that the neutrophil expression of PD-L1 potentially serves in some way to mitigate such release. Thus, neutrophil expression of PD-L1 would be serving in another anti-inflammatory role. However, whether this is a result of autocrine/ paracrine actions of PD-L1 expression among neutrophils leading to the suppression of the neutrophil’s typically inflammatory activities commonly seen/detected in response to the stimuli associated with shock/sepsis or is it a result of neutrophil expressed PD-L1 ligating a target on the vascular endothelial cell, possibly PD-1 (which an EC can express under inflammatory stressors, etc. [59]) remains to be established here.

### 2.4 Limitations.

Maybe the most overt limitation of such an approach is that the gene deficiency created in these Cre-Lox animals are constitutive relative to the cell-lineage effect being examined, so, it is not possible to consider the impact of gene deficiency in a temporal/post-insult fashion. Inasmuch, when considering the significance of these cell-lineage restricted impacts of gene deficiency we need appreciate the pre-dispositional nature of such a loss on the pathology here as opposed a more temporally specific/treatment-like inhibition. In this regard it is worth noting that while less cell-lineage selective in nature, Xu et al [31] use of liposomally encapsulated PD-L1 siRNA intra-venous delivery at 2 hours post-Hem/CLP to mitigate morbid effects of this dual insult indirect ARDS model supports the PD-L1 as a potential early (2–24 hours post-insult) temporal target. Also, as Xu et al [31] observed a reduction lung morbidity and several systemic plasma pro-inflammatory cytokines/chemokines (including the chemokine MCP-1 that we have reported was effected in this study) under these delivery conditions, it suggest the predominant pathological activity was likely also at the vascular endothelial cell interface and not so much due to its actions of PD-L1 expressing neutrophils.

## Conclusions

Taken together, these study results we have presented here support the hypothesis we have previously proffered that overexpression of PD-L1, specifically when upregulated on endothelial cells, has a morbid impact that would seem be due PD-L1 direct/indirect role in endothelial cell barrier function possibly mediated by MCP-1/CCL2 activity. Somewhat surprisingly we have also uncovered a potential immune protective role of PD-L1 expression on neutrophils, which our prior studies had not evidenced, suggesting that loss of such myeloid cell expression of PD-L1 was permissive anti-angiogenic and select inflammatory responses that may have a role in poorer mouse survival.

## Supplementary Material

Supplementary Files

This is a list of supplementary files associated with this preprint. Click to download.

• SuppltFig1ECPDL1deficentbreedB6.pdf

• SuppltFig2PMNPDL1deficentbreedB6.pdf

• SuppltFig3ADFlowConfirmPDL1defECorPMNCre.pdf

• SuppltFig4HemCLPiALISchematicCompatibilityMode.pdf

• SuppltFig5C57BLvControlSurvival.pdf

• SuppltFig6AGC57BLBALFlungCytosChems.pdf

• SuppltFig7AGC57BLPlasmaCytosASTALTBUN.pdf

## Data Availability

The datasets analyzed for this study can be found in the Brown Digital Repository [https://doi.org/10.26300/hq60-9z26].
